# The emotional attentional blink: what we know so far

**DOI:** 10.3389/fnhum.2013.00151

**Published:** 2013-04-23

**Authors:** Maureen McHugo, Bunmi O. Olatunji, David H. Zald

**Affiliations:** ^1^Vanderbilt Brain Institute, Vanderbilt UniversityNashville, TN, USA; ^2^Department of Psychology, Vanderbilt UniversityNashville, TN, USA; ^3^Department of Psychiatry, Vanderbilt UniversityNashville, TN, USA

**Keywords:** emotion, attentional blink, stimulus-driven attention, amygdala, anxiety

## Abstract

The emotional attentional blink (EAB), also known as emotion-induced blindness, refers to a phenomenon in which the brief appearance of a task-irrelevant, emotionally arousing image captures attention to such an extent that individuals cannot detect target stimuli for several hundred ms after the emotional stimulus. The EAB allows for mental chronometry of stimulus-driven attention and the time needed to disengage and refocus goal-directed attention. In this review, we discuss current evidence for the mechanisms through which the EAB occurs. Although the EAB shares some similarities to both surprise-induced blindness (SiB) and other paradigms for assessing emotion-attention interactions, it possesses features that are distinct from these paradigms, and thus appears to provide a unique measure of the influence of emotion on stimulus-driven attention. The neural substrates of the EAB are not completely understood, but neuroimaging and neuropsychological data suggest some possible neural mechanisms underlying the phenomenon. The importance of understanding the EAB is highlighted by recent evidence indicating that EAB tasks can detect altered sensitivity to disorder relevant stimuli in psychiatric conditions such as post-traumatic stress disorder (PTSD).

Rapid detection of emotionally salient events is critical for survival. However, given capacity limits on attention and awareness, the ability of emotional stimuli to preferentially capture attention comes with a cost. Specifically, if attentional resources are drawn to an emotional stimulus, there will be less processing capacity available for other stimuli. Although several different tasks have been used to explore the effects of emotional stimuli on spatial and selective attention, recent studies using more novel paradigms have begun to provide insights into the time course of attentional capture to emotionally salient stimuli and the impact of this capture on the ability to perceive subsequent stimuli. In this review, we discuss the emotional attentional blink (EAB) as a model paradigm for understanding stimulus-driven influences of emotion on attention. We contrast the EAB to other paradigms for studying emotion-attention interactions and review current neuroimaging and neuropsychological data for the mechanisms underlying the EAB. We conclude with a review of emerging evidence on the potential utility of the EAB as a measure of attentional biases to concern-relevant stimuli in psychopathology.

## Initial studies of the EAB

**Figure 1:**
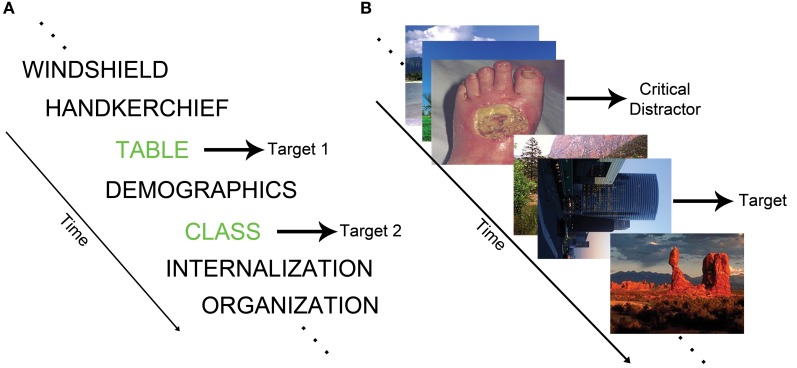
**(A)** Schematic of a standard AB task in which the goal is to report the identity of two words printed in green. **(B)** Example of an EAB trial in which participants must detect a rotated image in the presence of a disgust distractor.

The attentional blink (AB) paradigm measures the temporal capacity limits of attention (Dux and Marois, 2009). In the standard AB task, identification of a first target (T1) during a rapid serial visual presentation (RSVP) stream transiently impairs the ability to detect a second target (T2) (see Figure 1A). The refractory period during which T2 cannot be detected is labeled the AB. The EAB involves the presentation of task-irrelevant emotional distractors during an RSVP target detection task (see Figure 1B). In this paradigm, emotional distractors elicit an AB, even though the distractor stimuli are not targets (Arnell et al., 2004; Most et al., 2005). This type of emotion-induced AB indexes the ability of emotional stimuli to rapidly capture attention.

Most et al. (2005) provided an early description of the EAB, and coined the term emotion-induced blindness. On each trial, they had participants search for a single rotated image depicting a landscape or building within an RSVP stream of upright landscape or architectural photos. At 200 (lag 2) or 800 (lag 8) ms prior to the target, a distractor appeared consisting of either a negative, neutral, or scrambled image. Accuracy was impaired when a target followed a negative distractor at lag 2 relative to lag 8. Critically, performance was substantially worse following the negative distractors than the neutral distractors at lag 2. The EAB could not be easily accounted for by factors such as the color of the negative distractors because the emotionally salient distractors caused a robust AB relative to scrambled distractor images that were created by rearranging and blurring the negative images. Examination of the time course of the EAB indicates that the effect can be seen as early as lag 1 (Most and Junge, 2008), but declines substantially as one moves longer than lag 2, such that it is progressively weaker at lag 4 and lag 6. The effect is typically gone by lag 8, and indeed there may be a modest enhancement of target detection at lag 8 (Ciesielski et al., 2010). Overall, the length of the emotion-induced blindness is roughly similar to the standard AB, which typically lasts for approximately 200–500 ms (Raymond et al., 1992).

The EAB is not limited to negative or aversive images. In a number of studies, we have observed that erotica induce an EAB that is often larger than that produced by aversive images (Most et al., 2007; Ciesielski et al., 2010). Such findings suggest that arousal rather than valence is a critical feature in generating attentional capture. The effect also occurs with verbal stimuli. Some of the first published demonstrations of an EAB effect utilized a verbal RSVP task in which participants had to detect words printed in a specific color (Arnell et al., 2004, 2007; Mathewson et al., 2008). In this paradigm, emotionally arousing and taboo distractor words produced an AB relative to neutral words when a color target occurred soon after an emotionally arousing distractor word.

The current meaning or value of the distractor stimulus can modulate the ability of a stimulus to cause an EAB. Smith et al. (2006) demonstrated that aversively conditioned stimuli cause a blink and Piech et al. (2009) showed that current motivational state of the participant can modulate the extent of an EAB (e.g., food stimuli induced a greater EAB when subjects were fasting). These modulations of the EAB appear to be relatively modest compared to the large magnitude of attentional capture by aversive or erotic pictures, but may enable use of the EAB as a measure of dynamic changes in stimulus valuation. This sensitivity to changes in stimulus value suggests the potential utility of the EAB as an objective marker of the effects of therapeutic interventions in psychiatric conditions in which stimulus-reinforcer associations potently drive behavior.

## Comparison with other emotion-attention interactions

The EAB provides a unique measure of attentional capture by emotional stimuli. Although several paradigms have been commonly used to study emotion-attention interactions, none of these paradigms appear to measure the same phenomenon as the EAB, or if they do, they lack the robustness of the EAB effect. Methodologically, the most similar paradigm to the EAB involves a variant of the standard AB in which a T1 target is followed by an emotional stimulus presented as the T2 target. This variant allows examination of the extent to which emotional stimuli can break through the refractory period of the AB. Critically, emotionally salient T2s emerge from the standard AB window more readily than their neutral counterparts (Keil and Ihssen, 2004; Anderson, 2005; Milders et al., 2006). For instance, in AB studies by Anderson and Phelps (Anderson and Phelps, 2001; Anderson, 2005) in which subjects had to detect words written in a specific color, the T2 was more likely to be detected if it consisted of an emotionally salient word instead of a neutral word. This effect occurred even though the meaning of the emotional and neutral words was irrelevant to the instructed task, which only required subjects to attend to each word's color. This type of enhanced detection of emotional T2 stimuli has also been observed for emotional facial expressions, with highly anxious individuals showing enhanced detection of fearful vs. happy faces presented at T2 (Fox et al., 2005).

The preferential detection of emotional T2 stimuli in the standard AB and the ability of emotional stimuli to capture attention in the EAB indicate prioritized processing of emotional stimuli. However, the two paradigms differ in terms of the processes being measured. The standard AB with emotional T2 characterizes preferential target detection under a condition of limited attentional resources, whereas the EAB focuses on the impact of attentional capture on the processing of other stimuli. In particular, these two paradigms differ in the extent to which they depend on distinct types of attention. Although attention may be carved at many joints, a commonly accepted categorization divides attention into goal-directed (top-down) and stimulus-driven (bottom-up) attention (Egeth and Yantis, 1997; Corbetta and Shulman, 2002). Goal-directed attention allows us to voluntary select stimuli from the environment whereas stimulus-driven attention reflects the ability of highly salient items to capture attention. In the standard AB the emotional T2 stimulus is task relevant, and congruent with goal-directed attention to colored words. By contrast, the EAB has the hallmarks of a stimulus-driven, bottom-up engagement of attention in that attention is captured even though the emotional stimuli are task irrelevant. We see little evidence of a goal-directed ability to overcome the EAB effect even when people receive monetary rewards for good performance (accurately detecting targets), and regardless of subjects' evaluation of how hard they try to do the task (Most et al., 2007). Of note, prior exposure to and expectation of highly arousing emotional distractors does not eliminate their ability to capture attention (Arnell et al., 2007).

In considering other tasks that can be used to examine emotion-attention interactions, we note that most of these paradigms either reflect a preferential detection of emotional stimuli, or the ability of emotional stimuli to interfere with goal-directed attention. As such, the literature often parallels the divide between standard AB with emotional T2 and the EAB. For instance, several tasks including backward masking and continuous flash suppression provide instances in which emotional stimuli are preferentially detected. In backward masking, an emotional stimulus, typically a face, is presented very briefly and followed immediately by a masking stimulus such as a neutral face (Esteves and Ohman, 1993; Pessoa et al., 2005). Emotional expressions can be detected with even short presentation times (10–20 ms). Continuous flash suppression is a technique in which awareness for a stimulus presented to one eye is suppressed while visual noise is presented to the other eye (Tsuchiya and Koch, 2005). Suppressed fearful faces appear to reach awareness more readily than happy or neutral faces (Yang et al., 2007; Tsuchiya et al., 2009). These tasks differ from the EAB primarily in that they are not typically used to measure the effect of emotion on awareness for a subsequent stimulus.

By contrast, the emotional Stroop measures the extent to which emotional information interferes with processing of non-emotional features of stimuli. This variant of the classic Stroop effect examines the extent to which individuals are slower to name the color of emotional words than neutral words (Williams et al., 1996). There is an element of stimulus-driven attention in that the semantic meaning of the word interferes with attention to the color of the word even though word meaning is incidental to the task. To minimize this distraction, subjects must use top-down attentional control to overcome emotional interference. Because it measures the extent to which emotion interferes with task relevant processing, the emotional Stroop can be argued to have at least a superficial similarity to the EAB. However, the emotional Stroop paradigm differs from the EAB in three critical ways. First, there is never a failure to see the stimulus in the emotional Stroop paradigm because the distracting emotional information and the goal relevant stimulus are not spatially or temporally dissociable. Second, unlike the EAB, in which individual stimuli capture attention, emotional Stroop effects do not show an individual stimulus effect. That is, the emotional Stroop effect is seen when entire blocks of words are threat related, but is absent when emotional words and neutral words are presented in a mixed block (Algom et al., 2004). Algom and colleagues argue that this pattern of results reflects a generic slowing rather than a classic selective attention mechanism. Finally, at least to date, there is little evidence that the EAB can be overcome by the application of top-down control, although it may be possible to modestly alter the magnitude of the EAB based on knowledge about the target (Most et al., 2005).

The effects of emotional stimuli on attention have also been examined in the context of visual search tasks. In such tasks, an emotional target (e.g., spiders) is detected faster in an array of neutral images than a neutral image is detected in an array of emotional targets, especially at larger matrix sizes (Ohman et al., 2001). Typically, this is attributed to a pop-out effect for the emotional stimuli that leads to faster detection. However, it is also possible that a slowed detection of the neutral stimuli among a matrix of threat images is due to attentional capture caused by one or more of the threat images, which transiently disrupts the ability to perceive the neutral stimuli. Unfortunately, as typically applied by researchers (without the addition of baseline measures of performance in the absence of any emotional stimuli), it is unclear to what extent findings from the visual search task reflect speeded detection of emotional stimuli, interference in detection of neutral stimuli, or both (Lipp, 2006). In contrast, the EAB paradigm allows for independent measurement of the distinct effects of emotional and neutral distractors on target detection.

The dot probe task (Macleod et al., 1986; Mogg and Bradley, 1999) is another popular measure for assessing the effects of emotion on attention. The task measures the extent to which attention is drawn to or away from a spatial location where an emotional cue (typically a threat face) has occurred by measuring whether reaction times are faster when a target appears at a position congruent or incongruent to the cue. However, unlike in the EAB, there is no evidence that the emotional cue prevents awareness of the target. Rather the emotional cue only delays the detection of the target, and this delay is extremely brief, often at the level of 20 ms or less, and the delay is not always observed in non-clinical samples (Schmukle, 2005; Frewen et al., 2008). Thus, while the dot probe task may capture an emotional impact on attention, it appears too brief and weak to represent the same phenomenon captured by the EAB, which can last for 100 s of ms, and is seen consistently in the healthy young adult samples that we have studied thus far.

In sum, the EAB phenomenon differs conceptually from common emotion-attention task paradigms and allows measurement of attentional capture in a clear manner that is not contaminated by other aspects of responsiveness to emotional stimuli. Because the EAB is robust even within healthy (non-clinical) individuals, it is well suited for studying emotion-attention interactions and the neural substrates mediating these processes. Also of note, while emotional stimuli are task relevant in emotion-attention paradigms such as the standard AB, emotional Stroop, and visual search paradigms, they are not task-relevant in the EAB. This latter factor becomes important in determining precisely which aspects of attention are influenced by emotion.

## Attentional capture and stimulus-driven attention

As noted above, emotion-attention interactions can be considered in the context of goal-directed vs. stimulus-driven attention, with the EAB showing the characteristics of stimulus-driven attention. Corbetta and Shulman (2002) have proposed a model in which goal-directed and stimulus-driven attention depend on largely separable neural networks: a goal-directed dorsal frontoparietal attention network, including the frontal eye fields (FEF), and intraparietal sulcus (IPS) and a stimulus-driven ventral network that includes the temporoparietal junction (TPJ) and ventral frontal cortex [including the anterior insula (AI) and lateral frontal regions]. The standard AB is thought to primarily relate to capacity limits to goal-directed attention. It is critically dependent on attention being allocated to the first of two targets (T1, T2) during the RSVP stream. By contrast, when the T1 is to be ignored, the T2 is readily detected (Raymond et al., 1992). According to two-stage bottleneck models of the AB, all stimuli in the RSVP stream undergo an initial stage of perceptual and semantic processing (Chun and Potter, 1995). This stage has a high capacity to process stimulus representations in parallel. When target stimuli appear, they compete for a second stage, limited capacity process that enables awareness of the target. The first stage representations are weak and susceptible to decay: failure to detect T2 occurs if processing of the T1 in the limited capacity second stage doesn't complete before the stage 1 representation of T2 fades. Two-stage bottleneck models are supported by functional magnetic resonance imaging (FMRI) data. The correct detection of targets during the AB has been linked to activation of the dorsal frontoparietal attention network in concert with primary and higher order visual areas, whereas activation of sensory cortices alone does not appear to be sufficient for conscious report of targets (Marois et al., 2000, 2004; Gross et al., 2004; Shapiro et al., 2007; Williams et al., 2008).

Non-emotional, task-irrelevant distractors can also impair target detection during an RSVP stream, particularly if they share perceptual or conceptual features with a target (Folk et al., 2002; Barnard et al., 2004; Maki and Mebane, 2006). This “contingent attentional capture” may be viewed as a hybrid condition in which there is a goal-directed attention filter that allows certain stimuli to capture attention. Contingent attentional capture has been shown to recruit cortical areas consistent with the stimulus-driven attentional network in concert with the dorsal attention network (Serences et al., 2005), reflecting the dynamic interplay of top-down and bottom-up processing in this paradigm.

Asplund and colleagues have recently characterized attentional capture driven by irrelevant, non-contingent distractors during RSVP (Asplund et al., 2010a,b). They found that novel, unexpected distractors robustly impair target detection (termed surprise-induced blindness, or SiB), but this capture effect lasts for only one or two trials. This robust SiB effect was most apparent at a distractor-target stimulus onset asynchrony (SOA) of 390 ms, and was subject to rapid habituation across trials. Asplund et al. also identified a second variant of the SiB—at a shorter distractor-target SOA (130 ms) they detected a longer-lasting but weaker capture effect; target detection was impaired, but not to the extent it was during the first two trials with a 390 ms SOA. SiB differs from the standard AB not only in its dependence on a task-irrelevant stimulus, but it is also unaffected by placement of a blank immediately following the surprise stimulus (a condition that typically attenuates the standard AB). Neuroimaging evidence supports the notion that the robust form of the SiB is distinct from the standard AB. Activity in the TPJ, a region thought to be critical for reorienting attention in a stimulus-driven manner (Corbetta et al., 2008), is increased on trials in which surprise distractors capture attention (Asplund et al., 2010b). By contrast, this area is not commonly observed during more traditional AB tasks (Marois et al., 2004).

Mechanistically, the EAB shares more in common with SiB than either the standard AB or contingent attentional capture. SiB and the EAB occur despite the task-irrelevance of the critical stimulus. They also both appear to be relatively automatic, and largely outside of voluntary control. The persistence of EAB and the weaker form of the SiB may be similar as well: experiments using a verbal form of the EAB (Arnell et al., 2007) and the weak form of the SiB suggest a decline of the effect after ~100 trials. Whether the EAB using aversive or erotic pictures diminishes after a similar number of repetitions remains to be seen, as studies to date have generally used fewer than 100 emotional stimuli for a given class of stimuli. The EAB and SIB also are similar in terms of a lack of lag 1 sparing (Most and Junge, 2008; Asplund et al., 2010a). Lag 1 sparing is a feature often found in studies of the AB (and contingent attentional capture) in which the blink is decreased if presentation of the T2 occurs in the serial position immediately following the T1 (Chun and Potter, 1995). The precise cause for lag 1 sparing is a source of debate, but most explanations center on either the importance of a specific temporal relationship between T1 and T2 (approximately 100 ms) or on the characteristics of the post-T1 stimulus (Dux and Marois, 2009). Lag 1 sparing is conceptually important in the AB literature, as the ability to explain this sparing has proven critical in the evaluation of different models of the AB. The absence of lag 1 sparing in the EAB and SIB thus suggests that the EAB and SIB involve mechanisms that are at least partially distinct from that of the standard AB.

Despite their similar levels of endurance across trials the weaker form of the SiB and the EAB differ dramatically in their time course within a given trial. The lag-dependent time course of the EAB more closely resembles the AB. Beginning at lag 2, emotional distractors robustly capture attention and the effect gradually returns to baseline (Ciesielski et al., 2010). Additionally, SiB is more dependent upon contextual novelty. The weak lag 1 SiB disappeared when surprise distractors were presented as frequently as the non-critical distractors (Asplund et al., 2010a). However, the EAB still occurs when emotional critical distractors are just as likely to appear as neutral critical distractors (Arnell et al., 2007).

## Bottleneck model of the EAB

**Figure 2:**
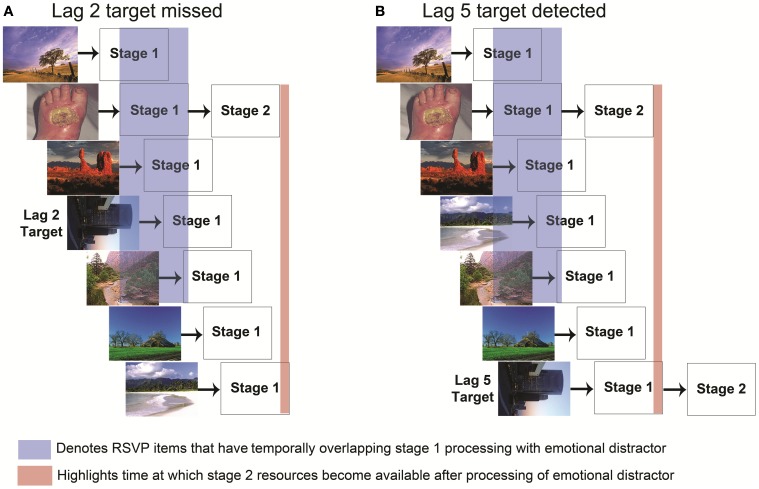
**Two stage bottleneck model of the emotional attentional blink with emphasis on stage 1 competition.** Each stimulus in the RSVP stream is processed by stage 1 perceptual resources in parallel. The time at which a stimulus enters stage 1 processing is determined by its presentation order in the RSVP stream. **(A)** If stage 1 processing of a task irrelevant emotional stimulus and the target overlap (highlighted in blue), their representations compete for selection into stage 2 processing. When the emotional stimulus is sufficiently strong (arousing), the strength of its representation combined with its appearance prior to T1 during the RSVP stream enables it to out-compete the target stimulus for entry into stage 2 processing. Despite being goal relevant, the representation of the target fades before the stage 2 processing of the emotional stimulus is completed (highlighted in red). **(B)** By contrast, if the target were to occur at a later lag, such as lag 5, there will be less competition due to the reduced temporal proximity to the emotional stimulus, and the target representation can last beyond the end of the stage 2 processing of the emotional stimulus (highlighted in red). In such a case the target would be able to enter stage 2 processing.

As noted previously, AB effects have often been explained with a two-stage bottleneck model (Chun and Potter, 1995) in which a target cannot be processed if the bottleneck stage is occupied with other processing. Although such a model could explain the EAB, in which an emotional distractor (like a T1 target) could occupy a second stage bottleneck, Most, Wang and colleagues (Most and Wang, 2011; Wang et al., 2012) have proposed an alternative possibility, in which emotional distractors generate increased competition for perceptual resources during stage 1 perceptual processing rather than limiting awareness at the central bottleneck stage (Figure 2). In this model, a robust representation of the emotional stimulus actively inhibits spatiotemporally adjacent goal relevant stimulus representations. Most and Wang (2011) hypothesized that if emotional distractors induce an EAB by creating competition for first-stage perceptual resources, the distractors should interfere with target processing primarily when the emotional distractor and target appear in the same spatial location. By contrast, if the distractor caused an EAB even when the target was at a different spatial location, it would suggest that the EAB occurred at a later, central processing bottleneck. To examine this possibility, they constructed a task in which participants searched for a single target in either of two simultaneously presented RSVP streams. Emotional and neutral distractors could appear in the stream containing the target or the other stream. Critically, the emotional distractors produced an EAB only when they were presented in the stream containing the target. These data are striking in that they suggest that the EAB does not depend on a single central bottleneck, but rather occurs at a spatially specific (and presumably) stage 1 processing level. This finding further suggests that the mechanisms underlying the EAB are at least partly dissociable from those of the standard AB: spatial selection is impaired during the AB (Jiang and Chun, 2001), whereas it appears to be largely intact during the EAB.

## Neural substrates of the EAB

Emotional stimuli elicit strong activation across the ventral visual stream (Sabatinelli et al., 2010) and this emotional modulation of visual processing is thought to be a key means by which emotionally salient items capture attention (Vuilleumier, 2005). The amygdala is robustly activated by emotional stimuli (Zald, 2003; Sergerie et al., 2008) and has been posited to enhance activation of higher order visual areas through its strong projections to visual regions (Amaral et al., 2003; Freese and Amaral, 2005). Support for the amygdala's role in such a process has been demonstrated for face processing in the fusiform gyrus (Vuilleumier et al., 2004), although it is possible that the amygdala is less critical for this modulation of other types of emotional stimuli.

If amygdala-driven persistence of the sensory representation of emotional distractors is a key factor in their ability to generate an EAB, there should be: (1) increased amygdala activity in response to emotional distractors when they capture attention, and (2) patients with amygdala lesions should exhibit a decreased EAB. At present, data directly supporting these two assertions remain lacking. Although a few studies have explored the neural correlates of the EAB with fMRI, to date, no study has specifically examined or reported amygdala activity in response to task-irrelevant emotional distractors when they do and do not capture attention. Most et al. (2006) investigated the extent to which amygdala activity in response to emotional distractors was influenced by top-down attentional settings. As expected, negatively valenced distractors elicited increased amygdala activity when presented during an RSVP stream similar to that used to study the EAB. However, the authors only examined activation on trials in which no target was presented, leaving the impact of this amygdala activation on attentional processing unclear. Neuropsychological data suggest that the amygdala may not be required for an EAB to occur. Our group recently examined whether patients with unilateral amygdala damage exhibit an EAB (Piech et al., 2011). We found that amygdala lesion patients, regardless of the side of the lesion, displayed comparable EABs to healthy controls for both negative and positive arousing distractors. Although it remains possible that the amygdala contralateral to the lesion was able to produce an EAB, the lack of even a mild decline in the frequency of the EAB following lesions to either hemisphere is striking. To rule out the possible compensation of the contralateral amygdala in producing an EAB, it would be helpful to determine whether patients with bilateral lesions show a similar preservation of the EAB. Such a preservation would be consistent with recent suggestions that there are multiple neural circuits by which emotional stimuli can influence attention (Pessoa and Adolphs, 2011).

An alternate hypothesis for understanding the mechanisms of the EAB is that emotional distractors capture attention by interrupting top-down attentional settings through interactions with the ventral attention network (Yamasaki et al., 2002) or by direct modulation of region in the goal-directed attention network (Pourtois et al., 2006). During attention tasks, the ventral stimulus-driven attention network is deactivated, which is thought to reflect a top-down filtering mechanism that helps suppress processing of information that is likely to be irrelevant to current goals (Corbetta et al., 2008). This suppression is interrupted by stimuli that attract attention. Findings from SiB experiments support this hypothesis: surprise distractors that generated a blink were linked not only to TPJ activity but also to activity in the inferior frontal junction (IFJ), a key locus in coordinating the interplay of stimulus-driven and goal-directed attention (Dux et al., 2006; Asplund et al., 2010b).

Despite the potential role of the TPJ in the SiB, it may not be critical for directing attention to emotionally salient events. The ventral attention network generally does not respond to salient, behaviorally irrelevant stimuli in a prolonged manner (Indovina and Macaluso, 2007; Corbetta et al., 2008), yet the EAB lasts for many trials. Indeed, in their study of the SiB, Asplund et al. (2010b) found that although surprise distractors elicited greater TPJ activity when they captured attention, both the behavioral index of attentional capture and TPJ activity rapidly habituated. Of note, while the amygdala and orbitofrontal cortex (OFC) responded to surprise distractors, they did not track attentional capture as measured by behavior. Given the divergence of the SiB and EAB as described above, additional work is needed to determine whether the TPJ plays a role in the EAB, and how this and other ventral attention regions interact with the amygdala and OFC in the presence of emotional distractors during RSVP.

Given the paucity of studies examining the neural substrates of the EAB, it is worth considering how findings from emotional variants of the standard AB task might relate to the EAB. A growing body of literature indicates that a network of regions including the amygdala, rostral anterior cingulate, thalamus, middle frontal cortex, and higher order visual areas contribute to the enhanced detection of emotional T2s during the AB window (Anderson and Phelps, 2001; Keil et al., 2006; De Martino et al., 2009; Lim et al., 2009; Padmala and Pessoa, 2010). The emotional standard AB finding most closely related to the EAB comes from a study by Schwabe et al. (2010) in which both T1 and T2 could be emotional or neutral. Critically, this manipulation allows assessment of activity both in response to emotional T1 that impair processing of subsequent T2 (somewhat analogous to the EAB) and emotional T2 that “break through” the AB. The authors found no evidence of amygdala activation when emotional T1 stimuli caused participants to miss T2 (either emotional or neutral). However, the AI, lateral OFC and dorsal anterior cingulate (dACC) were more active when an emotional T1 word impaired T2 identification compared to when T2 were correctly reported. These data suggest that anterior components of the ventral attention network such as the AI may play a more important role than posterior areas in orienting attention to distracting emotional cues and driving attention effects. Although the task is not a perfect match to EAB studies in that T1 was goal relevant, the absence of amygdala activation supports the possibility that extra-amygdalar neural circuits play a role in the EAB. Future FMRI studies are necessary to better understand whether the circuits involved in emotional target detection play a role in the EAB. Finally, emotional T2 detection during the standard AB is enhanced by increasing endogenous levels of norepinephrine (De Martino et al., 2008). This finding is particularly intriguing given a hypothesis proposed by Aston-Jones and Cohen (2005) that firing of norepinephrine-producing neurons of the locus coeruleus is responsible for the AB. It would be of interest to examine whether the EAB is similarly subject to noradrenergic modulation.

## Disengagement and delayed enhancement of attention

While the natural focus of the EAB paradigm is the disruptive effect of stimulus-driven attention, by measuring the length of time (lag number) at which individuals return to baseline performance (equivalent to neutral or no distractor trials), it is possible to obtain a metric of how long it takes an individual to disengage from an emotional stimulus and reassert top-down control of attention. Alternatively, in a study with just an early and a late lag, a disengagement efficiency index can be calculated by simply taking the difference in accuracy between an early and a late lag performance (e.g., lag 8 performance—lag 2 performance) (Olatunji et al., 2011b). In our past studies subjects show the largest disengagement efficiency for erotic images, reflecting a large blink at lag 2, but a strong ability to disengage and reassert top-down control at lag 8.

At long distractor-target delays, performance may reflect more than just disengagement. In the original studies of the EAB, lag 8 was treated as equivalent to baseline, and indeed there was no overall difference between neutral and aversive targets at this time point. However, in some samples, we and others have observed that performance is modestly improved following emotionally salient stimuli relative to neutral stimuli at lag 8 or longer lags, with notable individual differences in the magnitude of the effect (Bocanegra and Zeelenberg, 2009; Ciesielski et al., 2010). Bocanegra and Zeelenberg (2009) have referred to this type of late enhancement as emotional hypervision. They reason based on a two-stage bottleneck model that an emotional cue could trigger an enhancement during stage 1 processing that could allow for a facilitation of processing that carries over onto targets that are temporally far enough removed from the initial target (or distractor) so as to not be in competition. In other words, if such an enhancement at the stage 1 level lasts longer than the stage 2 bottleneck's refractory period, it will produce a period of improved detection at lags slightly longer than the typical length of the EAB effect. Bocanegra and Zeelenberg (2009) suggest that a single common source of emotional modulation could produce both the EAB and emotional hypervision effects through simultaneous influences on stage 1 and stage 2 processes. The parsimony of this model has appeal, as it requires only a single stimulus-driven process. There is however an alternative possibility in which hypervision effects during RSVP paradigms are not caused by the same mechanisms as the EAB, but rather reflect a distinct, independent source of stage 1 enhancement that is slower and longer lasting than the stage 2 bottleneck's refractory period. The relative slowness of the effect could reflect a multisynaptic pathway that requires more time for stimulus evaluation before it can modulate stage 1 perceptual processing. Regardless of the specific mechanism, assessment of individual differences in performance at intermediate and longer lags may provide useful information regarding the mental chronometry of emotion-attention interactions.

## EAB sensitivity and anxiety

Cognitive and neural models highlight the role of dysregulated attentional processes in the etiology of anxiety (Eysenck et al., 2007; Bishop, 2008) In a series of studies, we have used the EAB to measure the extent to which individuals with different anxiety disorders exhibit increased attentional capture or difficulty disengaging from concern-relevant stimuli. Recent data from patients with post-traumatic stress disorder (PTSD) is perhaps the most striking result from these studies. Attentional biases that automatically direct attention to trauma-relevant cues have been argued to play a key role in the maintenance of PTSD (Ehlers and Clark, 2000). Combined with a general hypervigilance, preferential attention to threat may lead to heightened fear responding to cues and repeated accessing of trauma-related memories. In a recent study employing the EAB paradigm, we observed that combat-exposed veterans with PTSD showed a powerful EAB for combat images relative to both healthy controls and combat exposed veterans without PTSD (Olatunji et al., 2012). Disgust and positive distractors evoked EABs in the PTSD veterans that were comparable to those observed in non-PTSD veterans and healthy controls, suggesting the absence of global hypervigilance. As discussed previously, the EAB is sensitive to current stimulus value (Smith et al., 2006). Future studies examining the extent to which the EAB is modulated following gold standard interventions for PTSD such as prolonged exposure therapy (Powers et al., 2010) would be informative.

By contrast, patients with obsessive compulsive disorder (OCD) have shown relatively normal levels of attentional capture at lag 2, but problems related to disengagement and the reestablishment of top-down attentional control at lag 8 (Olatunji et al., 2011b). This problem with disengagement emerged across emotional stimuli (reflected in a low disengagement efficiency index), but was most notable for erotic images. This may reflect a disorder specific concern (related to guilt or moral scrupulousness), or may reflect measurement sensitivity (since erotica consistently produces the most robust levels of attentional capture across samples, it provides the biggest challenge for disengagement mechanisms).

Patients with generalized anxiety disorder (GAD) displayed heightened attention to threat-related distractors at both short and long lags relative to healthy controls, consistent with elevated threat sensitivity (Olatunji et al., 2011a). However, the data from this study indicate that GAD is also associated with a reduced ability to recruit attentional control in response to neutral distractors, which may suggest the presence of a more general problem in attentional control that extends beyond the emotion domain. Indeed, in that study GAD patients reported significantly lower attentional control on a self-report measure, and the relationship between task performance for neutral stimuli and GAD diagnosis was shown to be mediated by attentional control as assessed by the Attention Control Scale (Derryberry and Reed, 2002).

## Conclusion

In summary, the EAB paradigm provides a robust and unique behavioral measure of the ability of emotional stimuli to preferentially capture attentional resources in a stimulus-driven manner. EAB effects can be characterized in relation to a two-stage bottleneck model of attention, and provide the ability to examine the mental chronometry of emotion-attention interactions. Data on the neural mechanisms of the EAB remain scarce, but current evidence suggests that the ventral frontoparietal attention network involved in stimulus-driven attention plays a critical role. Given the sensitivity of the EAB paradigm for detecting specific alterations in attentional capture and disengagement in anxiety disorders, the further delineation of the neural basis of the EAB may prove fruitful for identifying mechanisms underlying unique aspects of anxiety pathophysiology. Such research may lead to not only a better understanding of the neural correlates of psychopathological processes in these disorders, but could provide a useful biomarker for clinical treatment studies, especially those that explicitly attempt to alter attentional biases (Schmidt et al., 2009; Bar-Haim, 2010; Hakamata et al., 2010).

### Conflict of interest statement

The authors declare that the research was conducted in the absence of any commercial or financial relationships that could be construed as a potential conflict of interest.
